# Clinicopathological Significance and Prognostic Value of the Caveolae Constitutive Proteins Dynamin‐2 and Caveolin‐1 in Oral Squamous Cell Carcinoma

**DOI:** 10.1111/jop.13646

**Published:** 2025-05-26

**Authors:** Yoshiaki Kishikawa, Koroku Kato, Kyosuke Hakoda, Hayato Funaki, Hisano Kobayashi, Yutaka Kobayashi, Hiroki Miyazawa, Natsuyo Noguchi, Shuichi Kawashiri

**Affiliations:** ^1^ Department of Oral and Maxillofacial Surgery Kanazawa University Graduate School of Medical Science Kanazawa Japan; ^2^ Department of Oral and Maxillofacial Surgery Imizu Municipal Hospital Imizu Japan; ^3^ Department of Oral and Maxillofacial Surgery Komatsu Municipal Hospital Komatsu Japan; ^4^ Department of Oral and Maxillofacial Surgery Japan Community Health Care Organization Kanazawa Hospital Kanazawa Japan

**Keywords:** caveolin‐1, dynamin‐2, prognosis, progression, squamous cell carcinoma

## Abstract

**Background:**

We investigated the clinicopathological significance of the expression of two caveolae component proteins, dynamin‐2 (DNM2) and caveolin‐1 (CAV1), in primary tumors of patients with oral squamous cell carcinoma (OSCC).

**Methods:**

Immunohistochemical staining for DNM2 and CAV1 was performed on resected primary tumor specimens from 80 OSCC patients, and the individual expressions and combined expression status of these proteins were analyzed in relation to clinicopathological factors and prognosis.

**Results:**

We observed that the DNM2 expression was significantly correlated with the OSCC T‐classification and the stage, while CAV1 expression was significantly correlated with the mode of invasion and recurrence. Moreover, the combined DNM2/CAV1 expression status was significantly correlated with the T‐classification, stage, cell differentiation, and recurrence. In terms of overall survival, the CAV1‐positive patients had a significantly poorer prognosis compared to the CAV1‐negative patients, and the patients who expressed neither DNM2 nor CAV1 had a significantly better prognosis than those expressing either or both proteins.

**Conclusion:**

These results suggest that in OSCC, the expression of DNM2 is involved in tumor growth and the expression of CAV1 is involved in tumor invasion, and DNM2 and/or CAV1 expression affects the progression and prognosis of OSCC. The expressions of DNM2 and CAV1 may therefore be useful markers for OSCC progression and prognosis.

## 1 Introduction

Oral cancer accounts for 1%–3% of all carcinomas, and approx. 90% of oral cancer cases are oral squamous cell carcinomas (OSCC) [1]. OSCC affects > 400 000 people per year worldwide, and its mortality rate has remained largely unchanged for the last decades, with a 5‐year survival < 50% [2]. In Japan, according to statistics from the National Cancer Center, 23 671 cases of oral and pharyngeal cancer were diagnosed in 2019, and 7827 deaths due to these cancers occurred in 2020. A major OSCC research focus is the identification of prognostic factors, as this could lead to improved prognoses for individuals with OSCC.

Caveolae are small invaginations (50–100 nm in dia.) in the plasma membrane that are responsible for lipid uptake and signal transduction. The major constituent proteins of caveolae are dynamin‐2 (DNM2) and caveolin‐1 (CAV1), which are involved in endosome formation and the regulation of several parameters related to cancer progression, including cancer cell migration, metastasis, angiogenesis, and proliferation [3]. These caveolae‐constituting proteins cause morphological changes in the caveolae and create the tumor microenvironment [4].

DNM2 acts on intracellular signaling pathways to promote tumor cell survival and proliferation. This protein is also involved in the formation of actin‐based plasma membrane protrusions such as pseudopodia and invasive protrusions and thereby promotes the migration, invasion, and metastasis of the cancer cells [5]. The overexpression of DNM2 has been implicated in tumorigenesis in various carcinomas [6, 7, 8].

CAV1 functions as a scaffolding molecule for several signaling molecules, including epidermal growth factor receptor (EGFR), and its overexpression and tumor‐promoting activity are associated with metastasis and poor prognosis in bladder [8], lung [9], cervical [10], and other cancers. However, the expression of CAV1 is downregulated in breast cancer [11] and ovarian cancer [12], suggesting that CAV1 has a biphasic function.

DNM2 and CAV1 interact directly, mediating plasma membrane cleavage and resulting in endosome formation [13]. Although there have been reports on the association of DNM2 and CAV1 expressions with cancer cell biology in bladder cancer [8], to our knowledge there has been no investigation into the significance of the expression profiles of DNM2 and CAV1 in OSCC. In this study, we examined the expressions of DNM2 and CAV1 in clinical specimens of OSCC and investigated the clinicopathological significance and prognostic value of the expression profiles of these proteins.

## 2 Materials and Methods

### 2.1 Patients and Specimens

The subjects were 80 consecutive patients with primary OSCC who underwent surgical resection at the Department of Oral and Maxillofacial Surgery of Kanazawa University Hospital during the years 2013–2020. The 45 men and 35 women ranged in age from 36 to 90 years (mean 65.5 years). The Union for International Cancer Control (UICC) system (ver. 8) [14] was used for TNM classification. The World Health Organization (WHO) criteria [15] were used to determine the grade of tumor differentiation. The YK classification described by Yamamoto et al. [16] was used to assess the mode of tumor invasion. Ethical approval for the present study was obtained from the Ethics Committee of the Kanazawa University Graduate School of Medical Science, and all methods were performed in accordance with relevant guidelines and regulations (Approval No. 2016–301). Written informed consent was obtained from each patient.

### 2.2 Immunohistochemistry

We immunohistochemically stained tissue specimens extracted from the 80 patients' biopsies and surgeries to investigate the expressions of DNM2 and CAV1. The tissue specimens had been fixed in 10% neutral buffered formalin and embedded in paraffin. We examined 4‐μm‐thick sections from each specimen. We deparaffinized the paraffin‐embedded sections and treated them with Immunosaver (Fujifilm Wako, Osaka, Japan) dilution solution for 30 min in a warm bath for antigen retrieval. Endogenous peroxidase was blocked by treatment with 0.3% hydrogen peroxide in methanol for 30 min. Ten minutes of blocking with nonspecific goat serum was performed next, followed by overnight (8 ~ 12 h) incubation with primary antibodies at 4°C.

As primary antibodies, we used Anti‐Dynamin2 antibody (ab3457, anti‐rabbit polyclonal antibody, Abcam, Cambridge, MA) at a 1:100 dilution for the detection of DNM2 and Anti‐Caveolin‐1 antibody (#3238, anti‐rabbit polyclonal antibody, Cell Signaling Technology, Tokyo, Japan) at a 1:250 dilution for the detection of CAV1. Immunoreactive proteins were detected with the EnVision Horseradish Peroxidase (HRP) system (Dako, Kyoto, Japan). We used diaminobenzidine tetrahydrochloride to visualize the expression of DNM2 and CAV1, and the specimens were counterstained with hematoxylin and mounted with cover slides.

### 2.3 Evaluation of Staining

**FIGURE 1:**
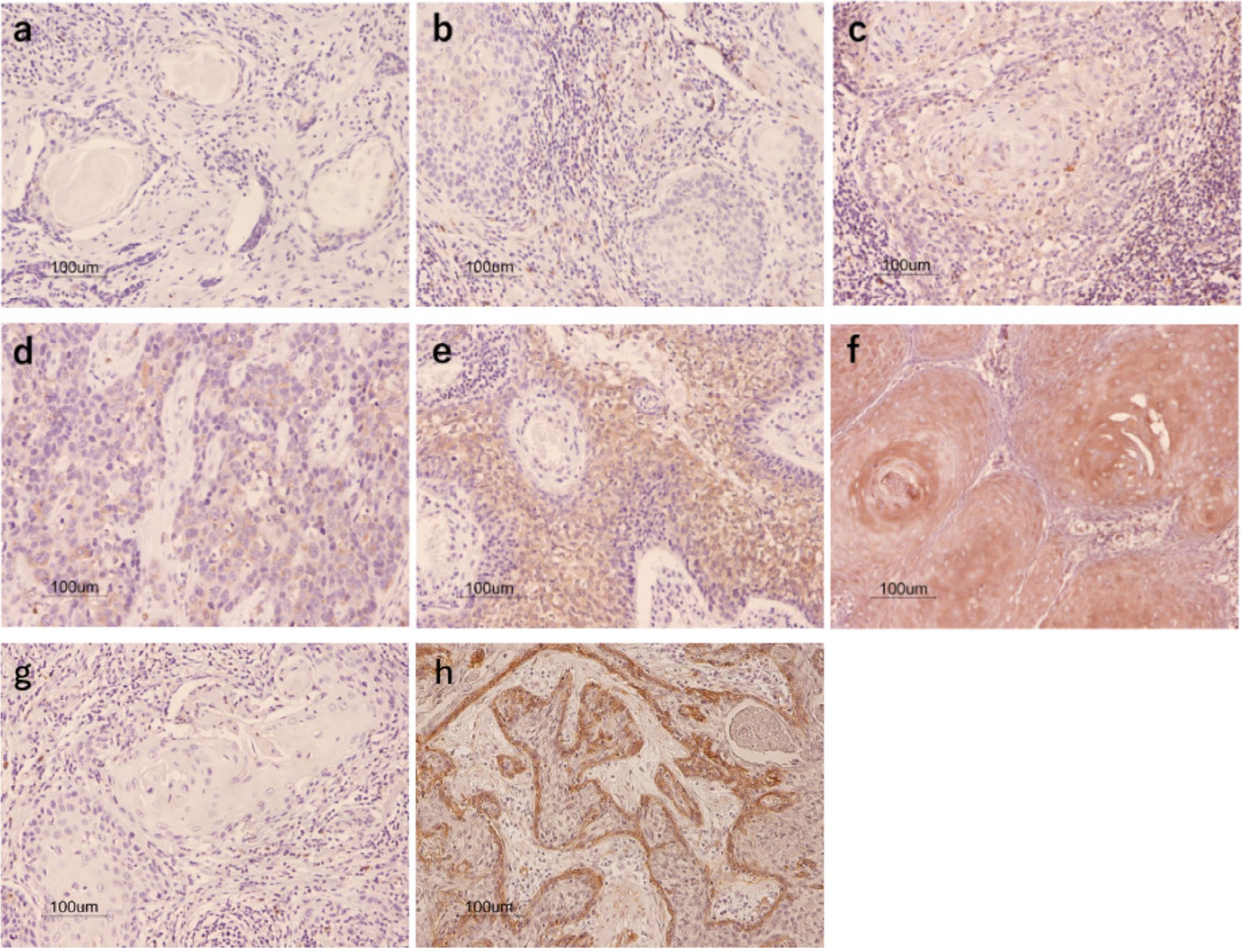
Immunohistochemical staining for DNM2 (a: PS0; b: PS1; c: PS2; d: PS3; e: PS4; f: PS5) and CAV1 (g, Negative; h, Positive). DNM2 was expressed mainly at the cytoplasm and, CAV1 was expressed mainly at the plasma membrane (original magnification ×100). PS: Proportion score.

Light microscopy (100× magnification) was used to examine the expressions of DNM2 and CAV1. Two reviewers (Y.K. and H.F.) who were blinded to all details of the tumors then calculated the percentage of positive cells by counting the number of positive cells among all tumor cells at the invasive front of the tumor in the specimen. A proportion score (PS) of 0–5 was then assigned to each specimen according to the percentage of positively stained cells: PS0 = no positively stained cells; (PS1 = < 1%; PS2 = 1%–10%; PS3 = 10%–33%; PS4 = 33%–66%; and PS5 = > 66%) (Figure 1). A receiver operating characteristic curve (ROC) was derived from the PS values, and specimens with a PS score ≥ 3 were deemed to be positive for DNM2 expression.

**FIGURE 2:**
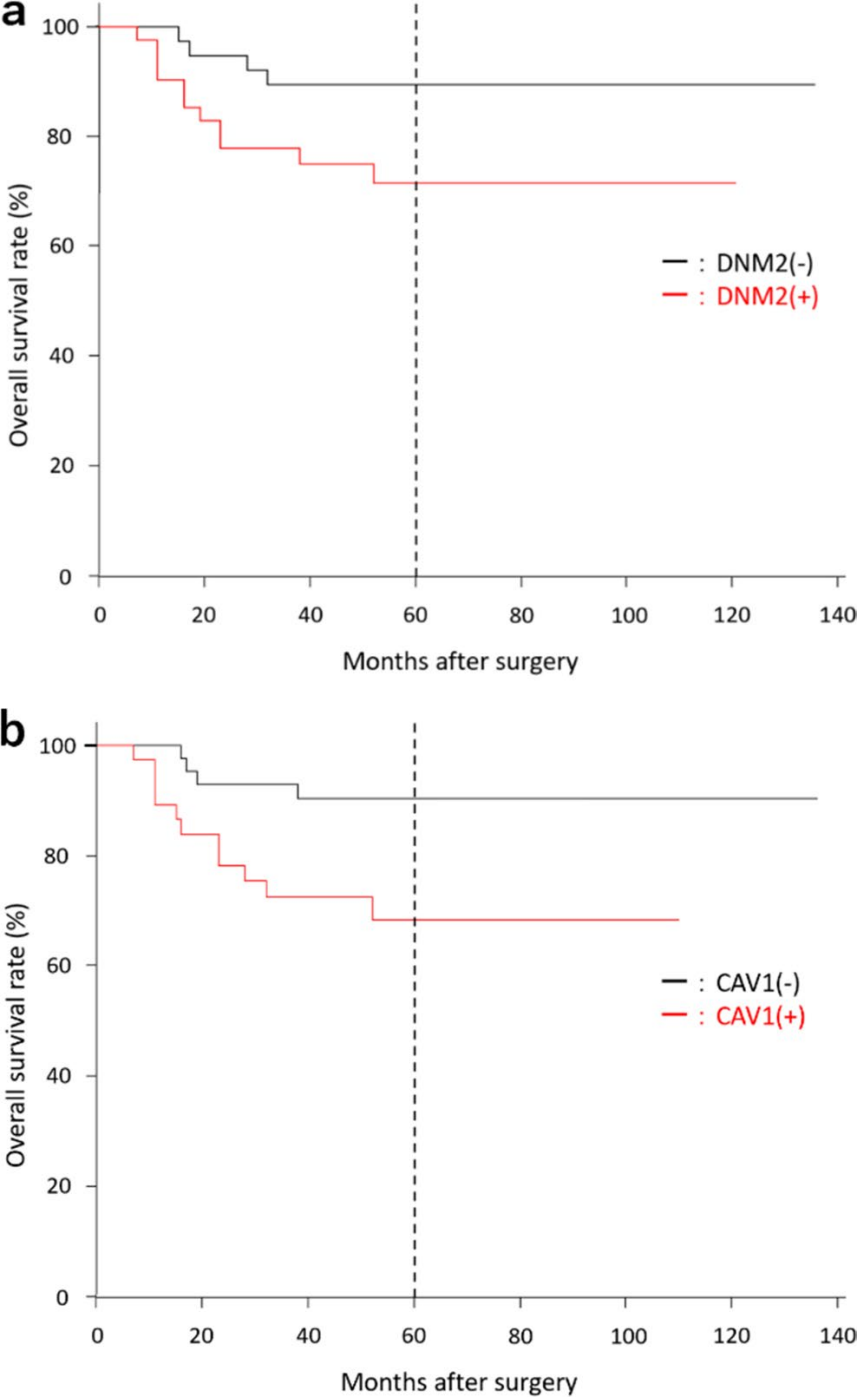
Kaplan–Meier curves showing the expression of (a) DNM2 and (b) CAV1 and overall survival.

The expression of CAV1 was determined in the same manner, but with a staining rate of ≥ 5% considered to indicate positive expression (Figure 2). We then evaluated the relation between the DNM2 and CAV1 expression profiles and the clinicopathological factors of age, sex, T classification, N classification, stage, cell differentiation, mode of invasion, depth of invasion (DOI), extra nodal extension (ENE), recurrence, and metastases. Finally, we assessed the prognostic significance of each factors based on the strength of its association with the DNM2 and CAV1 expression profiles.

### 2.4 Statistical Analyses

A Chi‐squared test was used to determine the relationship between DNM2 and/or CAV1 expression and each clinicopathological parameter. Overall survival was calculated using the Kaplan–Meier method and further evaluated by the log‐rank test. A multivariate analysis was performed using Cox's multivariate proportional hazards analysis for parameters that showed significant differences. Probability values < 0.05 were accepted as significant. The Easy R (EZR) software program (Saitama Medical Center, Jichi Medical University, Saitama, Japan) was used for the statistical analyses [17].

## 3 Results

The immunohistochemical analysis revealed that DNM2 was expressed mainly in the cytoplasm, and CAV1 was expressed mainly in the plasma membrane (Figure 1). The relationships between the DNM2 and CAV1 expressions and each of the clinicopathological factors are summarized in Table 1. DNM2 expression was correlated with the T classification, stageand DOI, and CAV1 expression was correlated with the T classification, mode of invasion, DOI, and recurrence. Table 2 shows the significance of the relation between the combined DNM2/CAV1 expression status and the clinicopathological factors. Significant correlations were observed between the combined DNM2/CAV1 expression status and the T classification, stage, cell differentiation, DOI, ENE, and recurrence.

**TABLE 1:** The relationship between the expression of DNM2 and CAV1 and clinicopathological factors.

			DNM2			CAV1		
		*n*	−	+	*p*	−	+	*p*
Age					0.242			0.083
	< 70	44	24	20		28	16	
	70≦	36	14	22		15	21	
Sex					0.955			1
	Male	45	22	23		24	21	
	Female	35	16	19		19	16	
T classification					< 0.001			0.046
	T1	19	17	2		15	4	
	T2	36	16	20		18	18	
	T3	17	3	14		8	9	
	T4	8	2	6		2	6	
N classification					0.709			0.659
	N0	61	28	33		34	27	
	N1	7	3	4		4	3	
	N2	12	7	5		5	7	
Stage					< 0.001			0.121
	I	17	15	2		13	4	
	II	26	11	15		14	12	
	III	18	3	15		9	9	
	IV	19	9	10		7	12	
Cell differentiation					0.512			0.614
	Well	37	15	22		21	16	
	Moderate	15	8	7		9	6	
	Poor	28	15	13		13	15	
Mode of invasion					0.860			0.015
	1	10	6	4		10	0	
	2	12	7	5		8	4	
	3	35	15	20		14	21	
	4C	17	9	8		8	9	
	4D	6	3	3		3	3	
DOI					0.014			0.004
	≦ 5	48	29	19		33	15	
	5 <, ≦ 10	18	6	12		6	12	
	10 <	14	3	11		4	10	
ENE					0.930			0.189
	−	77	36	41		43	34	
	+	3	2	1		0	3	
Recurrence					0.121			0.028
	−	61	32	29		37	24	
	+	19	6	13		6	13	
Metastasis					1			0.128
	−	43	20	23		27	16	
	+	37	18	19		16	21	

**TABLE 2:** The relationship between the expression aspects of DNM2 and CAV1, and clinicopathological factors.

			DNM2/CAV1				
		*n*	−/−	−/+	+/−	+/+	*p*
Age							0.182
	< 70	44	20	4	9	11	
	70 ≦	36	8	5	9	14	
Sex							0.916
	Male	45	17	5	9	14	
	Female	35	11	4	9	11	
T classification							0.002
	T1	19	14	3	1	1	
	T2	36	12	4	7	13	
	T3	17	2	1	6	8	
	T4	8	0	1	4	3	
N classification							0.533
	N0	61	21	6	16	18	
	N1	7	3	0	1	3	
	N2	12	4	3	1	4	
Stage							0.008
	I	17	12	3	1	1	
	II	26	9	2	6	9	
	III	18	3	0	6	9	
	IV	19	4	4	5	6	
Cell differentiation							0.028
	Well	37	12	2	10	13	
	Moderate	15	4	4	6	1	
	Poor	28	12	3	2	11	
Mode of invasion							0.243
	1	10	4	0	6	0	
	2	12	6	1	2	3	
	3	35	10	4	7	14	
	4C	17	6	3	2	6	
	4D	6	2	1	1	2	
DOI							0.024
	≦ 5	48	24	4	10	10	
	5 <, ≦ 10	18	3	3	3	9	
	10 <	14	1	2	5	6	
ENE							0.016
	−	77	28	7	18	24	
	+	3	0	2	0	1	
Recurrence							0.048
	−	61	26	5	12	18	
	+	19	2	4	6	7	
Metastasis							0.291
						
	−	43	15	4	13	11	
	+	37	13	5	5	14	

Next, we examined the association of protein expression as well as clinicopathological factors with overall survival (Figure 2). In regard to DNM2, the DNM2‐positive group had an overall survival rate of 73.8%, versus 89.5% for the DNM2‐negative group. Although the difference was not statistically significant (*p* = 0.055), the DNM2‐positive group showed a trend toward worse prognosis. In regard to CAV1, the CAV1‐positive group had an overall survival rate of 70.3% versus 90.7% in the CVA1‐negative group; this difference was significant (*p* = 0.018), indicating a correlation between CAV1 expression and prognosis.

**FIGURE 3:**
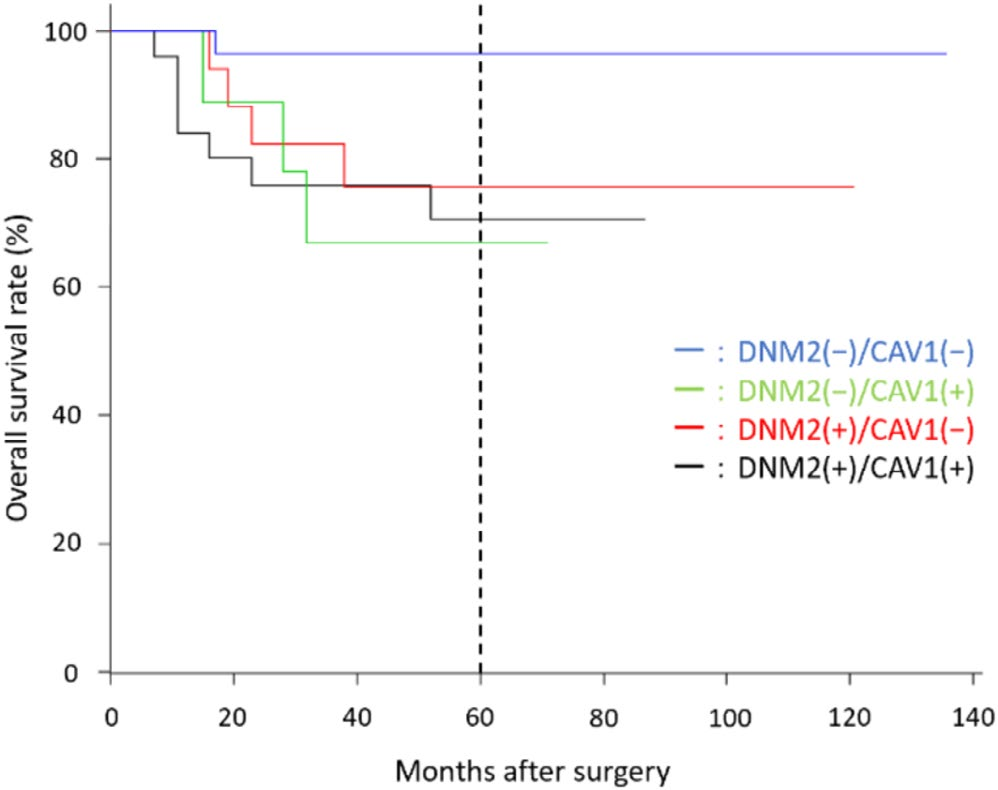
Kaplan–Meier curves showing the expression profiles of DNM2 and CAV1 and overall survival.

Finally, we examined the relation between the combined DNM2/CAV1 expression profile and overall survival (Figure 3). The overall survival rates were 96.4% in the DNM2−/CAV1− group, 66.7% in the DNM2−/CAV1+ group, 77.8% in the DNM2+/CAV1− group, and 72.0% in the DNM2+/CAV1+ group. There was no correlation between the expressions of DNM2 and CAV1, either singly or together, and prognosis (*p* = 0.067), but the DNM2−/CAV1− group had a better prognosis than the other groups.

A univariate analysis showed that age, mode of invasion, recurrence, metastasis, and CAV1 expression were significant prognostic factors, and the subsequent multivariate analysis demonstrated that only age, mode of invasion, and recurrence were independent prognostic factors for OSCC (Table 3).

**TABLE 3:** Univariate and multivariate analyses of clinicopathological factors and the expression and aspects of DNM2 and CAV1 in relation to overall survival in patients with oral squamous cell carcinoma (*n* = 80).

		Survivors	Non survivors	Log rank		Cox regression	
Variables	Groups	(*n* = 65)	(*n* = 15)	χ^2^	*p*	*p*	Hazard ratio
Age	< 70/70 ≦	38/27	6/9	4.0	0.046	0.020	5.09
Sex	Male/Female	35/30	10/5	0.5	0.459		
T classification	T1−2/3−4	44/21	10/5	0.1	0.762		
N classification	N−/N+	49/16	10/5	0.9	0.332		
Stage	I, II/III, IV	36/29	6/9	1.6	0.212		
Cell differentiation	Well/Moderate, Poor	33/32	4/11	2.8	0.092		
Mode of invasion	1, 2, 3/4C, 4D	53/12	4/11	18.4	< 0.001	< 0.001	56.99
DOI	≦ 5/5 <	45/20	3/12	20.9	< 0.001	0.590	
ENE	−/+	64/1	13/2	5.6	0.018	0.068	
Recurrence	−/+	56/9	5/10	18.8	< 0.001	< 0.001	47.12
Metastasis	−/+	39/26	4/11	5.4	0.020	0.201	
DNM2	−/+	34/31	4/11	3.7	0.055		
CAV1	−/+	39/26	4/11	5.6	0.018	0.078	
DNM2/CAV1	Others/(−/−)	38/27	14/1	6.7	0.009	0.973	

## 4 Discussion

Caveolae are flask‐like lipid rafts with narrow lumens of 50–100 nm that form invaginations in the plasma membrane; they are rich in proteins, cholesterol, and sphingolipids, and they are known to be involved in lipid uptake and endocytosis [18]. Changes to the characteristics of the caveolae have been shown to cause heterogeneity in the tumor metabolism of various cancers, supporting oxidative mitochondrial metabolism in cancer cells and contributing to the formation of the surrounding tumor microenvironment [19]. One of the main components of the caveolae is caveolin. In fact, a string‐like structure (a caveolae filamentous coat) of caveolin forms the entire cytoplasmic side of the caveolae [3]. Dynamin has been reported to be involved in the release of caveolae from the plasma membrane and to be involved in caveolae endocytosis [13].

The family of caveolins currently consists of CAV1, CAV2, and CAV3, each of which is encoded by three independent genes. CAV1 and CAV2 are abundant in type I lung cells, endothelial cells, fibroblasts, and adipocytes and are co‐expressed in the same cells and tissues, whereas CAV3 is expressed exclusively in myocytes (mainly skeletal and cardiac muscle cells) [4, 13]. Among the caveolins, CAV1 is an important component of caveolae and has been shown to have two opposing roles in tumor progression [4]. It acts as a tumor suppressor in the early stages of carcinogenesis, and at later stages, it is associated with tumor progression and metastasis. Regarding the expression of CAV1 in tumor cells, it has been alternately suggested that a high expression of CAV1 acts as a tumor‐promoting factor when expressed at high levels [20] and as a tumor‐suppressing factor when expressed at low levels [21]. These discrepant findings concerning the function of CAV1 thus merit further research.

Dynamin, which is involved in endocytosis along with caveolae, also exists in three different isoforms: DNM1, DNM2, and DNM3 [22]. Dynamins have been demonstrated to play a dual role in clathrin‐dependent endocytosis (CME), both regulating clathrin‐coated pit maturation at the early stages of CME and directly catalyzing membrane fission and clathrin‐coated vesicle formation at the later stages [23]. DNM2 is a large GTPase molecule that is consistently expressed in normal tissues [24] and is known to have a role in vesicle formation and intracellular membrane trafficking in endocytosis and also to function as a regulator of the actin and microtubule cytoskeleton [25]. In relation to cancer, it has been shown that DNM2 is required for the degradation of the extracellular matrix of invasive cancer cells [26] and for the endocytosis of several proteins involved in cancer motility and invasion [27].

DNM2 has also been reported to play an important role in vascular endothelial growth factor (VEGF) mediated angiogenesis [28] and to promote tumor cell invasion and metastasis by stabilizing different actin‐based structures involved in cell migration, remodeling the extracellular matrix, and degrading adhesion at the primary site [25]. Thus, although previous studies have suggested that the expressions of DNM2 and CAV1 affect cancer cell invasion and metastasis, to our knowledge, no study has evaluated the clinicopathological effects of these protein expressions in OSCC. We thus conducted the present study to investigate the expression profiles of DNM2 and CAV1 in patients with OSCC and their relation to the clinicopathological factors and prognosis.

In all 80 cases examined herein, DNM2 was expressed in the cytoplasm of cancer cells in the clinical specimens. In contrast, our results revealed that CAV1 was expressed only in the clinical specimens from some of the patients, and it was expressed on the plasma membrane of cancer cells. The observed localization of DNM2 and CAV1 in the OSCC calls seemed reasonable, since DNM2 is ubiquitously expressed in normal tissues and is involved in vesicle formation and intracellular membrane trafficking, while CAV1 is involved in the organization of caveolae at the plasma membrane.

The relationship between DNM2 expression and clinicopathological factors showed significant differences between DNM2 expression of tumor cells and the T classification and stage, DOI of OSCC. The DNM2+ cases had a poorer prognosis than the DNM2– cases, although the difference was not significant. DNM2 overexpression has been correlated with various factors impacting biological grade and prognosis—for example, cancer cell growth, migration, and invasion—in carcinomas of the breast [6], ovaries [7], and bladder [8]. Our present findings suggest that DNM2 is involved in primary tumor progression and prognosis in OSCC.

We observed that the expression of CAV1 was significantly associated with the T classification, mode of invasion (YK classification), DOI, and recurrence in OSCC. The YK classification is classified according to the pattern of cancer cell invasion and is considered an indicator of prognosis, with type‐4D cases in particular being highly invasive and having a poor prognosis. Hence, the CAV1+ cases had a significantly poorer prognosis than the CAV1– cases. It has been suggested that CAV1 is involved in the development and progression of many cancer types, including lung [9], cervical [10], and breast [11] cancers, as well as in the invasion of cancer cells [21, 29, 30]. In their study of squamous cell carcinoma of the tongue, Xue et al. reported that CAV1 expression increases in a stepwise fashion as the disease progresses from normal mucosa to hyperplastic, precancerous lesions, and squamous cell carcinoma [29]. It has also been reported that CAV1 expression is a poor prognostic factor in primary tumors and metastatic lymph nodes of OSCC [30]. The results of our present investigation suggest that CAV1 expression affects the tumor biological grade, particularly with respect to invasiveness.

In this study of DNM2 and CAV1 expression status and clinicopathological factors of OSCC, significant differences in T classification and stage, cell differentiation, DOI, ENE, and recurrence were identified among the four DNM2/CAV1 expression status groups. In relation to prognosis, no correlation was observed among the four profile groups, but the DNM2−/CAV1− group had a significantly better prognosis than the other groups (*p* = 0.009). In other words, the results suggest that the expression of either DNM2 or CAV1 affects the progression, biological grade, and prognosis of OSCC, while the combined DNM2/CAV1 expression profile was not a predictor of prognosis in OSCC in a multivariate analysis.

In conclusion, our results suggest that DNM2 and CAV1, the major components of caveolae, have distinct functions in OSCC. The results of our examination of the associations between the combined DNM2/CAV1 expression status and clinicopathological factors suggest that the formation and release of caveolae affect the progression, biological grade, and prognosis of OSCC. We thus propose that DNM2 and CAV1 are useful markers of the progression and prognosis of OSCC. The evaluation of these markers in OSCC could inform decisions regarding the indication for postoperative treatment, which in turn could improve prognoses.

## Author Contributions

Y.K. formulated the study and wrote the manuscript, and Y.K., K.H., and H.F. carried out the experiments. H.K. and Y.K. analyzed data, and Y.K. and H.M. wrote the manuscript. K.K., N.N., and S.K. contributed to the study planning, and K.K. carried out the data analysis and wrote the manuscript.

## Conflicts of Interest

The authors declare no conflicts of interest.

## Peer Review

The peer review history for this article is available at https://www.webofscience.com/api/gateway/wos/peer‐review/10.1111/jop.13646.

## Data Availability

The data that support the findings of this study are available on request from the corresponding author. The data are not publicly available due to privacy or ethical restrictions.
